# Analysis of Bias in Measurements of Potassium, Sodium and Hemoglobin by an Emergency Department-Based Blood Gas Analyzer Relative to Hospital Laboratory Autoanalyzer Results

**DOI:** 10.1371/journal.pone.0122383

**Published:** 2015-04-07

**Authors:** Jian Bo Zhang, Ji Lin, Xiao Dong Zhao

**Affiliations:** 1 Emergency Department, First Affiliated Hospital of Chinese PLA General Hospital, Beijing 100048, China; 2 Institute of Basic Medicine, Chinese PLA General Hospital, Beijing 100853, China; Azienda Ospedaliero-Universitaria Careggi, ITALY

## Abstract

**Objective:**

The emergency departments (EDs) of Chinese hospitals are gradually being equipped with blood gas machines. These machines, along with the measurement of biochemical markers by the hospital laboratory, facilitate the care of patients with severe conditions who present to the ED. However, discrepancies have been noted between the Arterial Blood Gas (ABG) analyzers in the ED and the hospital laboratory autoanalyzer in relation to electrolyte and hemoglobin measurements. The present study was performed to determine whether the ABG and laboratory measurements of potassium, sodium, and hemoglobin levels are equivalent, and whether ABG analyzer results can be used to guide clinical care before the laboratory results become available.

**Materials and Methods:**

Study power analyses revealed that 200 consecutive patients who presented to our ED would allow this prospective single-center cohort study to detect significant differences between ABG- and laboratory-measured potassium, sodium, and hemoglobin levels. Paired arterial and venous blood samples were collected within 30 minutes. Arterial blood samples were measured in the ED by an ABL 90 FLEX blood gas analyzer. The biochemistry and blood cell counts of the venous samples were measured in the hospital laboratory. The potassium, sodium, and hemoglobin concentrations obtained by both methods were compared by using paired Student’s *t*-test, Spearman’s correlation, Bland-Altman plots, and Deming regression.

**Results:**

The mean ABG and laboratory potassium values were 3.77±0.44 and 4.2±0.55, respectively (*P*<0.0001). The mean ABG and laboratory sodium values were 137.89±5.44 and 140.93±5.50, respectively (*P*<0.0001). The mean ABG and laboratory Hemoglobin values were 12.28±2.62 and 12.35±2.60, respectively (*P* = 0.24).

**Conclusion:**

Although there are the statistical difference and acceptable biases between ABG- and laboratory-measured potassium and sodium, the biases do not exceed USCLIA-determined limits. In parallel, there are no statistical differences and biases beyond USCLIA-determined limits between ABG- and laboratory-measured hemoglobin. Therefore, all three variables measured by ABG were reliable.

## Introduction

Depending on the severity and nature of the illness, biochemical markers such as liver function markers, electrolytes, and blood gases must be measured in some patients who present to an emergency department (ED). For example, it is important to measure the electrolyte and hemoglobin levels in older patients, patients with renal dysfunction, and patients who are taking diuretics. In addition, the patients with severe anemia are waiting for the blood transfusion treatment and those with hyperkalemia need reduce the serum potassium level immediately. Such biomarker information can be quickly obtained by point-of-care testing (POC) with an arterial blood gas (ABG) analyzer [1]. Thus emergency management can be quickly performed. However, studies have shown that POC test measurements are not consistent with hospital laboratory measurements of the same samples [2]. As a result, many clinicians do not trust or utilize the electrolyte and hemoglobin measurements of ABG analyzers, although they do value the ventilation and acid–base status data provided by ABG analyzers.

Recently, our hospital in China was equipped with the ABL 90 FLEX blood ABG analyzer. The potassium, sodium, and hemoglobin values ABG-measured should be reliable according to the clinical observation, albeit they were lower than those laboratory-measured. To further investigate the measurement’s reliability using ABG analyzer, we carried out the present prospective observational study.

## Materials and Methods

All enrolled patients were older than 18 years, presented to the ED of the First Affiliated Hospital of Chinese PLA General Hospital between May, 2013 and Dec, 2013, and, due to their illnesses, routinely provided paired arterial and venous blood samples that underwent ABG, full blood cell count, and biochemical marker analyses. The study was approved by the Ethics Committee of the hospital. The Ethics Committee required that all enrolled patients give verbal consent to participate in the study. All consecutive patients (or their relatives) who agreed to having their measurements recorded and analyzed were enrolled. Their agreement was also recorded in both the patient medical records and the experimental records.

For study power analyses, a pilot study with 50 consecutive patients was performed to determine the bias of ABG analyzer results for potassium, sodium, and hemoglobin relative to laboratory results. The pilot study revealed that a sample size of 195 patients would be sufficient for detecting significant differences between ABG- and laboratory-measured hemoglobin levels (Table 1). Smaller samples sizes were needed to detect significant differences between ABG- and laboratory-measured potassium (n = 6) and sodium (n = 3) levels. Thus, a total sample size of 200 consecutive patients was established and an additional 150 consecutive patients who met the eligibility requirements were enrolled. Among those 204 patients, 4 patients with missing values or with the mistaken paper report, rather than with the result out of the biological plausible ranges, were excluded from the analysis. Ultimately, 200 patients were left for the statistical analysis. The blood gas samples were taken by experienced nurses in the ED, all of whom had been trained to follow the same arterial puncture method. The arterial samples were measured by the ED-located ABL 90 FLEX blood gas analyzer (Radiometer Medical ApS, Copenhagen, Denmark) within 3 minutes of the blood being taken. Venous blood samples were taken within 30 minutes of the arterial blood sampling and sent to the hospital laboratory. The biochemical markers were measured by a vt-5600 automatic biochemical analyzer (Johnson & Johnson Services, Inc. New Brunswick, New Jersey, USA) while the full blood cell counts were measured by a XT-1800i automated hematology analyzer (Sysmex Corporation, Kobe, Japan). All instruments involved in this study were maintained regularly according to the product manual.

**Table 1:** Determination of study sample size.

	n*	Mean bias (Laboratory-ABG)	SD	95%LOA	Estimated sample size
Potassium (mmol/L)	50	0.433	0.394	0.32–0.54	6
Sodium (mmol/L)	50	3.5	2.215	2.87–4.13	3
Hemoglobin (g/dL)	50	0.192	1.078	−0.11–0.5	195

Abbreviations: ABG, arterial blood gas; LOA, limit of agreement; SD, standard deviation.

The sample sizes were calculated with the formula in the medical statistics textbook as follows.

$$\text{n=}{\left[\left({\text{u}}_{\text{α}}{\text{+u}}_{\text{β}}\right)\text{σ/δ}\right]}^{\text{2}}$$

The following picture was taken from the textbook.

- α was determined 0.05, β was determined 0.20. u_0.05_ = 1.6449(single side). U_0.20_ = 0.8416. σ means SD of mean bias of the two data groups. δ means mean bias of the two data groups.

For example, sample size of hemoglobin was:

$${\left[\left(1.6449+0.8416\right)\times 1.078÷0.192\right]}^{2}\approx 195$$

Fifty paired arterial and venous blood samples were used in a pilot study to determine the mean biases for use in study power calculations.

To elucidate the statistical difference, the paired ABG analyzer and laboratory potassium, sodium, and hemoglobin data were analyzed and the laboratory measurement was considered the gold standard. To reduce potential bias caused by deviation of potassium, sodium, and hemoglobin levels from a normal distribution, we applied the natural-log transformation to normalize the distributions of all data before the statistical analysis. Lastly, we used paired Student’s t’-test, Spearman’s correlation, Bland-Altman plots, and Deming regression. All statistical analyses were performed by using Medcalc analyze software version 11.4.2.0 (MedCalc Software, Acacialaan 22, B-8400 Ostend, Belgium). P values of <0.05 were considered to indicate statistical significance.

## Results

Totally 204 pairs of samples were enrolled to this study and 4 pairs of samples were excluded. All the 4 reasons for exclusion were errors in reports of ABG analyzer.

**Fig 1:**
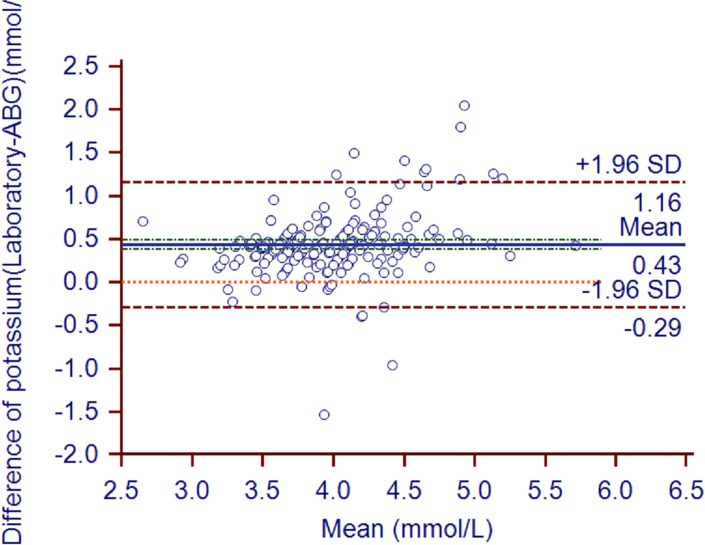
Potassium bias plot. Bland and Altman plot shows the potassium bias of each pair of samples (in mmol/L). The solid line indicates the mean bias and the value is 0.43. The green line indicates 95%CI of mean bias (0.38 to 0.48). The brown line indicates the 95% LOA of the bias (-0.29 to 1.16). The orange line indicates zero line.

**Fig 2:**
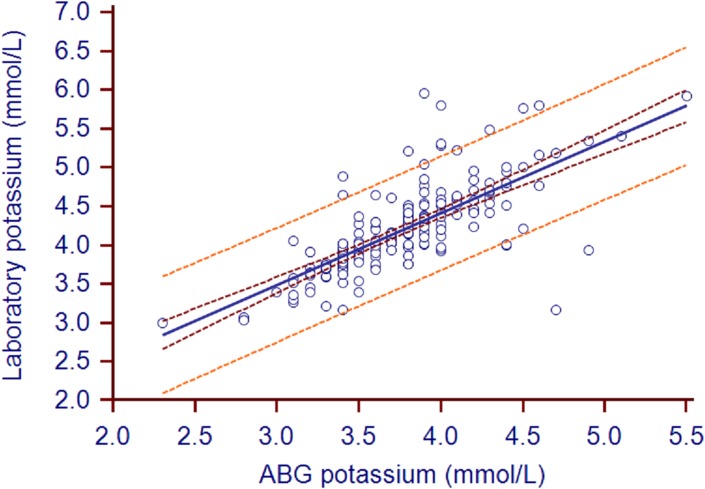
Potassium scatter plot with Deming fit. Concordance plot shows agreement between laboratory- and ABG-measured potassium (in mmol/L). The solid line indicates the correlation coefficient (r = 0.74). The brown line indicates the 95%CI of correlation coefficient (0.67 to 0.8). The orange line indicates the 95%LOA.

The mean ABG analyzer and laboratory potassium values were 3.77±0.44 and 4.2±0.55, respectively (*P*<0.0001; Table 2). The mean bias (95% confidence intervals) was 0.43 (0.38–0.48) mmol/L (Fig 1). The biases in 44 pairs of values surpassed the limits of USCLIA (0.5 mmol/L). The correlation coefficient (95% confidence intervals) between the two types of potassium measurements was 0.74 (0.67–0.8). The Deming fit between the paired samples is shown in Fig 2.

**Table 2:** Comparison of the potassium, sodium, and hemoglobin results obtained by the arterial blood gas analyzer and the hospital laboratory.

	Mean±SD(Laboratory)	Mean±SD(ABG)	*P*	Mean bias(95%CI)	Sample numbers surpassed the limits of USCLIA	LOA of difference	Correlation coefficient (r) (95%CI)	Deming regression equation
Potassium (mmol/L)	4.2±0.55	3.77±0.44	<0.0001	0.43 (0.38–0.48)	44	−0.29 to 1.16	0.74 (0.67–0.8)	0.73+0.92x
Sodium (mmol/L)	140.93±5.50	137.89±5.54	<0.0001	3.04 (2.73–3.34)	32	−1.24 to 7.31	0.92 (0.9–0.94)	14.70+0.92x
Hemoglobin (g/dL)	12.35±2.60	12.28±2.62	0.24	0.08 (-0.05–0.21)	21	−1.77 to 1.92	0.94 (0.92–0.95)	0.94+0.93x

Abbreviations: ABG, arterial blood gas; CI, confidence intervals; LOA, limit of agreement; SD, standard deviation.

The P value showed statistical difference(P<0.05) of the two groups of results. The LOA of difference showed 95% LOA of the bias. The correlation coefficient and 95%CI showed to what extent the two groups of results related. The deming regression equation showed in what way the two groups of results related.

**Fig 3:**
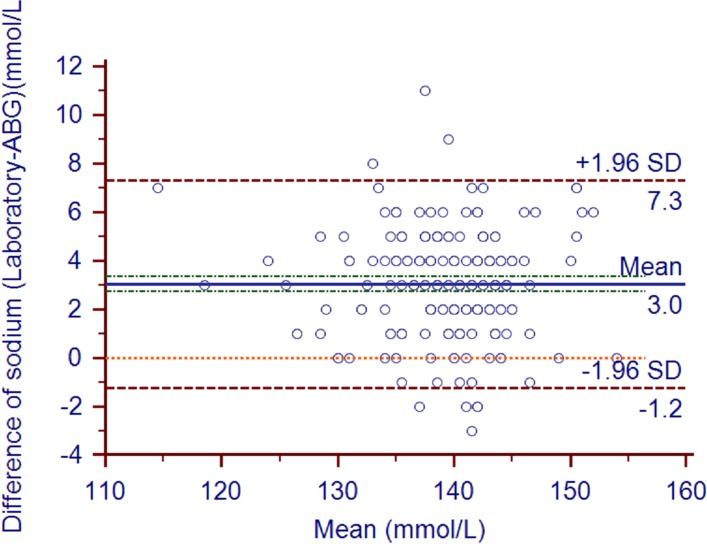
Sodium bias plot. Bland and Altman plot shows the sodium bias of each pair of samples (in mmol/L). The solid line indicates the mean bias and the value is 3.04. The green line indicates 95%CI of mean bias (2.73 to 3.34). The orange line indicates the 95% LOA of the bias (-1.24 to 7.31). The orange line indicates zero line.

**Fig 4:**
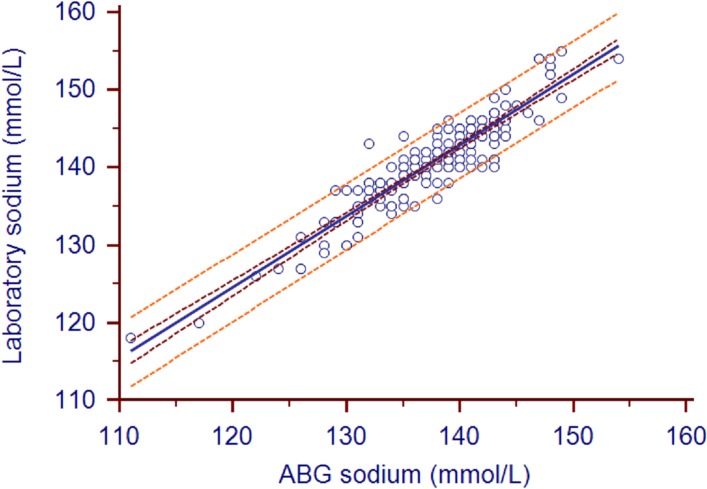
Sodium scatter plot with Deming fit. Concordance plot shows agreement between laboratory- and ABG-measured sodium (in mmol/L). The solid line indicates the correlation coefficient (r = 0.92). The brown line indicates the 95%CI of correlation coefficient (0.9 to 0.94). The orange line indicates the 95%LOA.

The mean ABG analyzer and laboratory sodium values were 137.89±5.44 and 140.93±5.50, respectively (*P*<0.0001). The mean bias (95% confidence intervals) was 3.04 (2.73–3.34) mmol/L (Fig 3). The biases in 32 pairs of values surpassed the limits of USCLIA (±4mmol/l). The correlation coefficient (95% confidence intervals) between the two types of sodium measurements was 0.74 (0.67–0.8). The Deming fit between the paired samples is shown in Fig 4.

**Fig 5:**
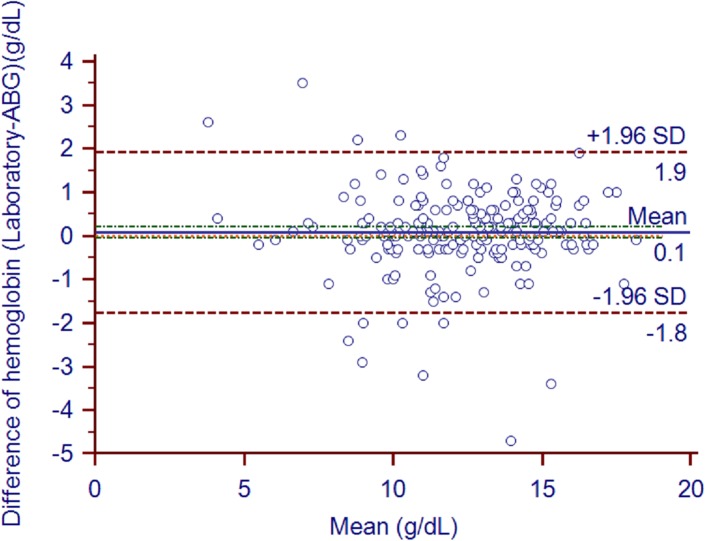
Hemoglobin bias plot. Bland and Altman plot shows the hemoglobin bias of each pair of samples (in g/dL). The solid line indicates the mean bias and the value is 0.08. The green line indicates 95%CI of mean bias (-0.05 to 0.21). The orange line indicates the 95% LOA of the bias (-1.77 to 1.92). The orange line indicates zero line.

**Fig 6:**
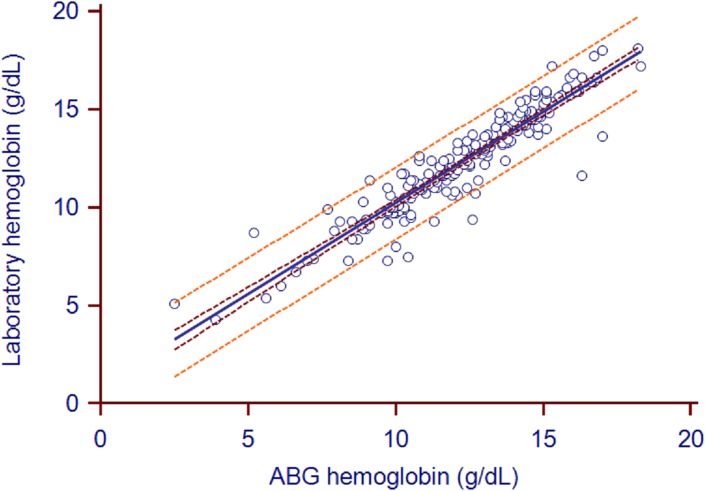
Hemoglobin scatter plot with Deming fit. Concordance plot shows agreement between laboratory- and ABG-measured hemoglobin (in g/dL). The solid line indicates the correlation coefficient (r = 0.94). The brown line indicates the 95%CI of correlation coefficient (0.92 to 0.95). The orange line indicates the 95%LOA.

The mean ABG analyzer and laboratory hemoglobin values were 12.28±2.62 and 12.35±2.60, respectively (*P* = 0.24). The mean bias (95% confidence intervals) for ABG-measured hemoglobin relative to laboratory-measured hemoglobin was 4% (1–2%)(Fig 5). The biases in 21 pairs of values surpassed the limits of USCLIA (±7%). The correlation coefficient (95% confidence intervals) between the two types of hemoglobin measurements was 0.94 (0.92–0.95). The Deming fit between the paired samples is shown in Fig 6.

## Discussion

Other hospitals and institutions have examined whether POC testing yields different results to laboratory testing. Indeed, it has been shown that even when identical analyzers, identical methods, and the same study population are used, POC testing yields different results for sodium and potassium [3]. The study by Chhapola *et al*., in which blood gas samples were processed with liquid sodium heparin, revealed that the sodium and potassium POC measurements showed a significant systematic bias and wide limits of agreement with laboratory values [4]. These authors suggested that these biases are not clinically acceptable. Yip *et al*. suggested that the heparin in the syringes may be affecting POC results because it raises the total volume of the sample and dilutes the plasma portion of the sample [5]. Indeed, in the present study, BD preset blood gas syringes containing solid Ca2+-balanced lithium heparin were used for sampling the arterial blood, as this is our routine practice. Compared to the biases reported by Chhapola *et al*., we found less wide biases for both sodium and potassium. In Chhapola’s study, the biases of potassium and sodium were 0.75mmol/L and 8.76mmol/L. However, there may also be additional reasons, such as the electrolyte-balanced heparin in blood gas syringes, for POC bias in terms of electrolyte measurements [6]. Other authors have suggested that the bias relates to the fact that the POC and laboratory tests involve two different machines, which necessitate the use of different methods and sample types [3][7]. Specifically, POC machines (like the ABL90 FLEX) process whole blood while the biochemical autoanalyzers in hospital laboratories employ serum.

According to the US Clinical Laboratory Improvement Amendment (USCLIA) [8], potassium, sodium, and hemoglobin biases that are within ±0.5 mmol/l, ±4mmol/l, and ±7%, respectively, are acceptable. In the present study, the hemoglobin levels measured by ABG and the laboratory did not differ significantly, the mean bias was within 4%, and the correlation coefficient was 0.94. Thus, the ABG-measured hemoglobin levels coincided well with the laboratory-measured values. By contrast, the ABG-measured potassium levels were on average 0.43 mmol/L lower than the laboratory measurement. While this was within the USCLIA cut-off of ±0.5 mmol/l, the 95% limits of agreement were −0.29 and 1.16. Thus, the ABG measurements of potassium were less accurate than the ABG hemoglobin measurements. While these ABG measurements of potassium may still be useful for assisting clinical judgments if the potassium value was significantly high or low, especially in patients who are prone to potassium abnormalities (*e*.*g*., due to renal failure or dysfunction), they cannot be used for administering reducing serum potassium or rapid potassium infusions but the bias should be considered. The ABG measurements of sodium were similarly inaccurate, although they again were within the acceptable margins set by USCLIA. However, since conditions that associate with sodium abnormality are relatively mild, this inaccuracy may be tolerable. Our position is that when ABG results reveal obviously high or low potassium and/or sodium levels, these findings can be used to guide clinical care, as this prevents time being wasted waiting for the more accurate laboratory results. However, once the laboratory results become available, the treatment should be checked and adjusted if necessary.

According to the product manual of the ABL90 FLEX blood gas analyzer, 17 variables in a blood sample can be measured within 35 seconds. By contrast, to measure serum electrolyte levels, time is lost transporting the sample to the hospital laboratory, centrifuging the sample, and waiting for the autoanalyzer results. In our hospital, the average time needed to obtain laboratory electrolyte data is 1.5 hours. This could be extended to 2 to 3 hours if the sample must be retested due to suspicion that the first result was anomalous. Moreover, the ABL90 FLEX blood gas analyzer requires as little blood as 65 μL, which is much less than is needed for routine tests in the hospital laboratory. This makes this ABG analyzer particularly useful for neonates [9]. Similar POC testing instruments have the same advantages. Thus, overall, the present study indicated that the ABL90 FLEX had good precision. Other POC testing instruments have also been shown to produce clinically acceptable results [10].

Although we evaluate the reliability of ABG analyzer’s measurement in this study, there are some limitations to the study. The first is that the number of the patients enrolled in the study is not too big despite of its enough power for the statistical analysis. We predict that the solid result will be provided with when more individuals are enrolled. Another limitation is that we do not perform the follow-up study for the patients enrolled in the study. If so, we can provide the proper and immediate treatment to the relative patients after we get ABG analyzer’s measurement.

## Conclusion

Generally, ABG-measured potassium, sodium and hemoglobin were lower than those of laboratory-measured. ABG-measured potassium and sodium had statistical difference with those of laboratory-measured. But the mean biases did not exceed USCLIA-determined acceptable biases. ABG-measured hemoglobin had no statistical difference with that of laboratory-measured. The bias was more lower and also did not exceed USCLIA-determined acceptable bias. All the three variables are somewhat reliable.
